# Evidence-based practice, research utilization, and knowledge translation in chiropractic: a scoping review

**DOI:** 10.1186/s12906-016-1175-0

**Published:** 2016-07-13

**Authors:** André E. Bussières, Fadi Al Zoubi, Kent Stuber, Simon D. French, Jill Boruff, John Corrigan, Aliki Thomas

**Affiliations:** School of Physical and Occupational Therapy, Faculty of Medicine, McGill University, Montréal, Canada; Département chiropratique, Université du Québec à Trois-Rivières, Trois-Rivières, Canada; Centre de Recherche Interdisciplinaire en Réadaptation (CRIR), Montréal, Canada; Division of Graduate Education and Research, Canadian Memorial Chiropractic College, Toronto, Canada; School of Rehabilitation Therapy, Faculty of Health Sciences, Queen’s University, Kingston, Canada; Schulich Library of Science and Engineering, McGill University, Montréal, Canada; The Canadian Chiropractic Guideline Initiative, Saskatoon Saskatchewan, Canada; Center for Medical Education, Faculty of Medicine, McGill University, Montréal, Canada

**Keywords:** Chiropractic, Scoping review, Evidence-based practice, Research utilization, Knowledge translation

## Abstract

**Background:**

Evidence-based practice (EBP) gaps are widespread across health disciplines. Understanding factors supporting the uptake of evidence can inform the design of strategies to narrow these EBP gaps. Although research utilization (RU) and the factors associated with EBP have been reported in several health disciplines, to date this area has not been reviewed comprehensively in the chiropractic profession. The purpose of this review was to report on the current state of knowledge on EBP, RU, and knowledge translation (KT) in chiropractic.

**Methods:**

A scoping review using the Arksey and O’Malley framework was used to systematically select and summarize existing literature. Searches were conducted using a combination of keywords and MeSH terms from the earliest date available in each database to May 2015. Quantitative and thematic analyses of the selected literature were conducted.

**Results:**

Nearly 85 % (56/67) of the included studies were conducted in Canada, USA, UK or Australia. Thematic analysis for the three categories (EBP, RU, KT) revealed two themes related to EBP (attitudes and beliefs of chiropractors; implementation of EBP), three related to RU (guideline adherence; frequency and sources of information accessed; and perceived value of websites and search engines), and three related to KT (knowledge practice gaps; barriers and facilitators to knowledge use; and selection, tailoring, and implementation of interventions). EBP gaps were noted in the areas of assessment of activity limitation, determination of psychosocial factors influencing pain, general health indicators, establishing a prognosis, and exercise prescription. While most practitioners believed EBP and research to be important and a few studies suggested that traditional and online educational strategies could improve patient care, use of EBP and guideline adherence varied widely.

**Conclusion:**

Findings suggest that the majority of chiropractors hold favourable attitudes and beliefs toward EBP. However, much remains to be done for chiropractors to routinely apply evidence into clinical practice. Educational strategies aimed at practicing chiropractors can lead to more EBP and improved patient care. The chiropractic profession requires more robust dissemination and implementation research to improve guideline adherence and patient health outcomes.

**Electronic supplementary material:**

The online version of this article (doi:10.1186/s12906-016-1175-0) contains supplementary material, which is available to authorized users.

## Background

Evidence-based practice (EBP), research utilization (RU), and knowledge translation (KT) are interrelated concepts that pertain to the identification, utilization and application of knowledge from research sources to clinical practice. EBP has been defined as “the integration of clinical expertise, patient values, and the best research evidence into the decision making process for patient care” [1]. RU is a sub-set of EBP, which refers to “that process by which specific research-based knowledge is implemented in practice” [2]. KT, on the other hand, emphasizes the synthesis, dissemination, exchange and application of knowledge from research findings, and from other sources, to influence changes in practice and improve health outcomes [3]. Thus, KT aims to help bridge the gap between research findings and what is routinely done in practice. Although there have been an increasing number of KT activities in recent years, much remains to be done to effectively translate research findings targeting healthcare professionals, consumers, and other stakeholders into clinical practice. However, one important initial step is to determine what is known about EBP, RU and KT among healthcare professionals.

A number of articles have been published on EBP and determinants associated with the use of evidence in different healthcare professions, including medicine [4–7], nursing [8, 9], dentistry [10], physical therapy [11–14], and occupational therapy [13, 15]. However, to date this area has not been reviewed comprehensively in the chiropractic profession [16]. Chiropractic is a regulated health profession serving approximately 10 – 15 % of the population annually [17]. Several barriers to implementing evidence in chiropractic practice have been previously proposed. These include: 1) limited research capacity, for example, less than 1 % of members of the Canadian chiropractic profession conduct research; 2) negative attitudes of clinicians towards research; 3) the need to fully implement broad-based EBP content in chiropractic training programmes; 4) the large percentage of chiropractors in solo practice, limiting opportunities to interact with colleagues and other professions; 5) the limited exposure to using decision support systems (for example, clinical decision rules, guidelines, etc.); 6) the lack of coordination of efforts between researchers, practitioners and stakeholders to successfully disseminate and implement guidelines; and 7) ongoing debates about the chiropractic profession’s own identity and related contrasting approaches (experiential vs. evidence-based) [16]. This has resulted in a wide range of attitudes and beliefs about EBP among chiropractors [18]. It is important to identify the evidence available on this topic in the chiropractic profession to ascertain successful KT and RU strategies in the profession. This may help determine the best methods of dissemination of clinical practice guidelines (CPGs) and other forms of evidence-based information most likely to yield successful outcomes for patients.

The purpose of this scoping review was to identify studies reporting on RU, KT, and EBP in chiropractic in order to provide a synthesis of information about barriers and enablers to the uptake of evidence into chiropractic practice, and inform a KT research programme to help close the ‘research-practice gap’ [19]. Specifically, the primary objective was to document the current state of knowledge on EBP, RU, and KT in the chiropractic profession and to determine themes that support each of these three categories. A secondary objective was to report factors that support the integration and/or utilization of research and other forms of evidence in chiropractic practice. A third objective was to formulate recommendations for the conduct of future KT research in chiropractic.

## Methods

Due to the uncertain volume of existing literature on evidence-based practice (EBP), research utilization (RU), and knowledge translation (KT), a scoping review methodology was preferred considering it is a flexible yet comprehensive approach to examining these topics. This methodology is optimal for answering our research question given that the breadth of information on the topic is unknown, and may arise from disparate sources and levels of evidence. In addition, scoping reviews set the scene for a future research agenda by documenting what is already known, and by using a critical analysis of gaps in knowledge to help refine research questions, concepts and theories [20, 21]. We used an a priori protocol based on the Arksey and O’Malley framework [22, 23] to address each of the five suggested steps for undertaking a scoping review:

### STEP 1: Identifying the research question

The research question that guided this scoping review was: *What is known about EBP, RU, and KT in chiropractic practice?* [22].

### STEP 2: Identifying relevant studies

A health sciences librarian (JB) conducted searches in EMBASE, CINAHL, MEDLINE, Index to Chiropractic Literature (ICL), PubMed publisher content, AMED and the Cochrane Library from the earliest date available in each database to May 28^th^ 2015 using a combination of keywords and MeSH terms. The search strategy was developed for MEDLINE and a modification of this strategy was used to search the other databases [see Additional files 1. MEDLINE search strategy used to identify research articles]. The search strategy was peer reviewed by another health sciences librarian using the Peer Review of Electronic Search Strategies checklist [24]. We further refined the search strategy iteratively with input from investigators and collaborators, and in consultation with an experienced librarian. The reference lists of all included articles were reviewed to ensure that no relevant articles were missed. The grey literature (organization websites, theses/dissertations, conference proceedings) was also searched by hand, electronically, and by contacting specific authors. The team librarian executed all final searches, exported the results into EndNote and removed all duplicates from the search results.

### STEP 3: Study selection

#### Inclusion and exclusion criteria

For primary studies, we included quantitative, qualitative and mixed methods studies that examined the use of evidence in chiropractic clinical practice, including the aspects/factors related to the person (knowledge, skills, attitudes, practice style, etc.) and the organization (culture, system, frameworks) that support and promote the use of evidence. For secondary studies, syntheses of existing evidence, theory, and reviews were included but narrative reviews and editorials or commentaries were excluded.

#### Screening

Prior to commencing the screening process, a calibration exercise was conducted to ensure reliability in correctly selecting articles for inclusion. This involved independently screening a random sample of 5 % of the included citations by two reviewers (research assistants (RAs)). Eligibility criteria were applied, and if low agreement was observed between the reviewers (e.g., a kappa statistic less than 0.50), a third reviewer (AB) was available to discuss and resolve discrepancies.

#### Agreement

Regarding study selection, we proceeded as follows: The RAs and the first author reviewed abstracts of all studies included in the first search. Following a first round of decisions regarding which papers should be excluded, two team members (KS and AT) reviewed a randomly selected subset (25 %) of abstracts. Percent agreement (between the first author and two team members not involved in the first selection round) was used as a measure of inter-rater reliability. Previous work in this area suggested that it would be reasonable to expect agreement between 80-90 % with a clearly defined research question and inclusion/exclusion criteria [15].

### STEP 4: Charting the data

A pre-defined data charting form recorded the following information for each study: author, year of publication, country, study design, purpose of the study/research question, clinical setting, population characteristics (e.g., years in practice, type of practice), methodology, whether the study dealt with EBP and/or RU and/or KT, intervention, outcome measures, results, implications for practice, limitations, and directions for future research. The charting form was tested independently by two RAs on a random sample of 10 articles and revised iteratively by the study team while the search was completed. Differences in data charting were resolved by discussion or with the involvement of a third reviewer (AB). The three reviewers met to determine whether their approach to data extraction was consistent with the research question and purpose. No formal quality assessment of included studies was performed as the aim of this scoping review was to identify the breadth of the literature and the major areas of research activity with corresponding resulting themes [See Additional files 2. Data Abstraction Table].

### STEP 5: Collating, summarizing and reporting the results

We synthesized the data according to three distinct steps: (1) Analysing the data (descriptive numerical and qualitative thematic analyses), (2) reporting the findings, and (3) discussing results implication [22].

#### Analysing the data

- *Descriptive numerical analysis:* The numerical analysis highlighted the nature and distribution of the studies (number of studies, study design, year of publication, country, study population, methodology and area of practice).
- *Qualitative thematic analysis:* The research question, study purpose and major findings were the primary units of analysis. We extracted the primary research questions common across the studies that met the inclusion criteria and identified the major themes emerging from the findings with a focus on EBP, RU, and KT in chiropractic practice. Two research team members (AB, KS) coded the data using a deductive approach to examine the categories and subsequent themes emerging from the units of analysis. Summarizing the results was an iterative process and as such, once the themes were generated, two other investigators (AT, SF) were involved in a discussion of the emerging themes using the charting tables. The first author (AB) went back to all the charting tables to confirm that they corresponded to the themes that had been generated. A summary of the major findings organized under each theme was produced following several iterations. To facilitate reporting the results of this review, we classified the included studies, based on their objectives and research questions, into three major categories, namely EBP, RU and KT [See Additional files 3. Thematic analysis of data].

#### Reporting the findings and producing the study outcome

We generated themes and reported both the qualitative and quantitative results in a table. The qualitative thematic analysis included nested concepts or categories that illustrated the themes.

## Results

### Descriptive numerical analysis

**Fig. 1:**
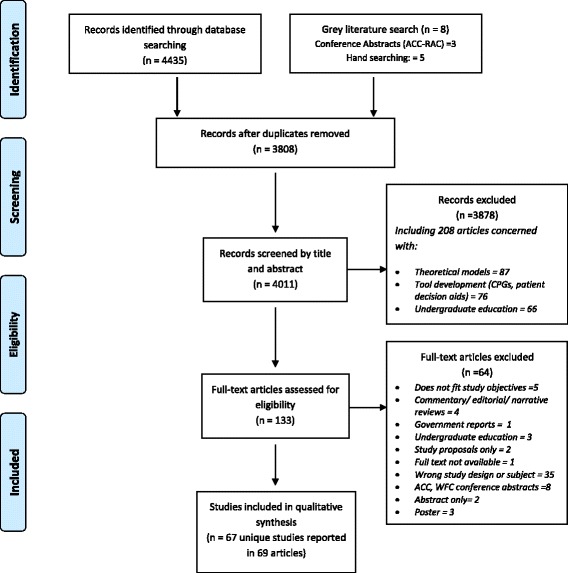
Results of search strategy and process of selecting research articles. Flow diagram describing the process of searching and selecting articles on research utilization, evidence-based practice and knowledge translation in chiropractic to be included in the scoping review

The search conducted up until May 28^th^ 2015 yielded 4443 citations, including 5 articles from hand searches of key articles and 3 articles from conference abstracts. A total of 4011 articles remained after removing duplicates. Initial screening of titles and abstracts resulted in the rejection of 3878 articles that did not meet the inclusion criteria. Articles on theoretical models (*n* = 87), tool development (*n* = 76) and undergraduate education (*n* = 66) were excluded at this stage. The remaining 133 full-text articles were assessed for eligibility by the first author and a group of four review authors who underwent an inter-rater reliability process to ensure agreement. The level of agreement between the authors was 75 %. Sixty-two full-text articles were excluded at this stage. A PRISMA flow chart was used to track the number of studies at each stage of the review (Fig. 1).

**Fig. 2:**
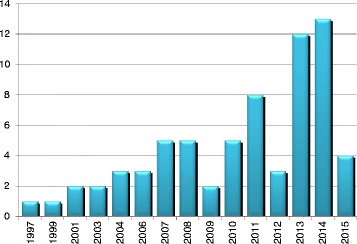
Articles by year of publication. Number of articles published each year between 1997 and May 2015 on research utilization, evidence-based practice and knowledge translation in chiropractic (*n* = 69)

**Fig. 3:**
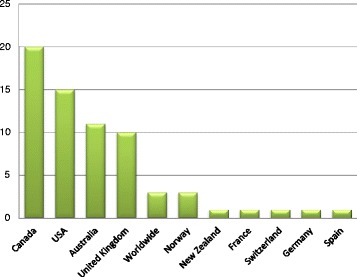
Studies by country of origin. Number of studies conducted in various countries around the world on research utilization, evidence-based practice and knowledge translation in chiropractic (n-67)

**Fig. 4:**
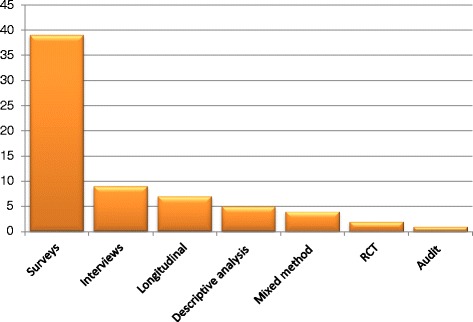
Studies by study design. Number of studies by types of study designs for all admissible studies (*n* = 67) on research utilization, evidence-based practice and knowledge translation in chiropractic

**Fig. 5:**
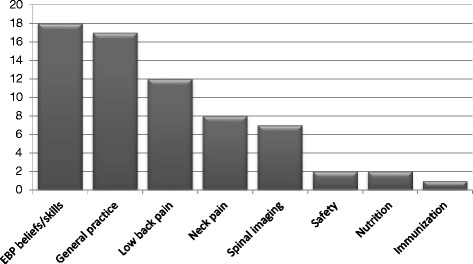
Studies by area of practice. Number of studies by area of clinical practice of included studies (*n* = 67)

**Fig. 6:**
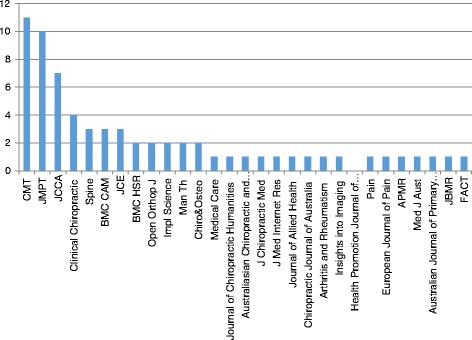
Studies by research category. Number of articles published in peer reviewed journals

Sixty-seven studies reported in 69 articles were included in the review. Sixty-five studies (94 %) were published between 2001 and May 2015. The number of studies increased steadily each year with the exception of 2009 and 2012 (Fig. 2). Over a third (*n* = 24) of articles were published in the years 2013 and 2014 alone. Canada produced the most studies (*n* = 20), followed by the US (*n* = 15), Australia (*n* = 11) and the UK (*n* = 10) (Fig. 3). Most of the studies used self-administered survey questionnaires (*n* = 39) or interviews (*n* = 9), descriptive analysis or mixed methods (*n* = 9). The remaining ten studies used quantitative methods, including longitudinal studies (*n* = 7), randomized controlled trials (RCTs) (n = 2), and one audit of clinical practice (Fig. 4). Based on area of clinical practice, the studies focused on: general chiropractic practice (*n* = 17), research beliefs and skills (*n* = 18), low back pain (*n* = 12), neck pain (*n* = 8), spinal imaging (*n* = 7), nutrition (*n* = 2) and immunization (*n* = 1) (Fig. 5). Authors affiliation with chiropractic educational institutions were as follows (represented by at least one author): CMCC (*n* = 9 articles), Palmer (*n* = 7), Anglo-European (*n* = 5), Murdoch (*n* = 5), UQTR (*n* = 4), other institutions (*n* = 1) including: NYCC, Welsh Institute of Chiropractic College, IFEC (Paris), University of Southern Denmark, Logan, Parker, University of Western States, Northwestern, Cleveland, RMIT, Swiss Institute, National). Of interest, the top three journals where included articles were published were Chiropractic and Manual Therapy (CMT, *n* = 11), closely followed by the Journal of Manipulative Physiological Therapeutics (JMPT, *n* = 10), and the Journal of the Canadian Chiropractic Association (JCCA, *n* = 7) (Fig. 6).

### Qualitative thematic analysis

**Fig. 7:**
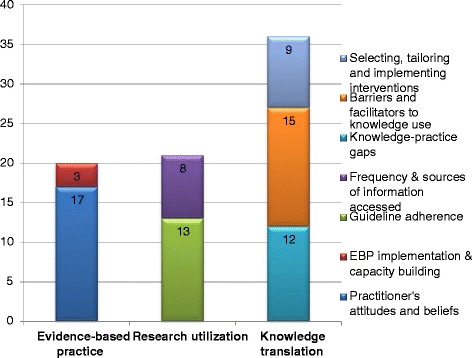
Studies by research category. Number of studies within respective categories (evidence-based practice, research utilization and knowledge translation) and corresponding themes

Included studies were classified into three major categories, namely evidence-based practice (EBP), research utilization (RU), and knowledge translation (KT). Each category was further sub-classified into major themes as follows: two themes under EBP (*attitudes and beliefs of chiropractors and implementation of EBP*), two under RU (*guideline adherence; frequency and sources of information accessed*) and three under KT (*knowledge practice gaps; barriers and facilitators to knowledge use; and selection, tailoring, and implementation of KT interventions*) (Fig. 7).

### Reporting the findings and producing the study outcome

I. Evidence-based practice (EBP)

Twenty of the 67 studies (30 %) were related to EBP and reflected two major themes: I) Attitudes and beliefs towards EBP (*n* = 17 articles) and II) Implementation of EBP (*n* = 3).

#### Theme 1: Practitioners’ attitudes towards, and beliefs, about of EBP

##### Sub theme 1: Philosophical beliefs and scope of practice

Varying perspectives about divergent attitudes toward chiropractic practice were reported in six studies [18, 25–29]. An earlier study suggested that Canadian chiropractors held views that fell into three categories: conservative (18.6 %), liberal (22 %), and moderate views (59.4 %) [25]. Recently, a representative sample of Canadian chiropractors reported views that could be categorized into six strata [18]. A minority (~19 %) of respondents continue to hold a predefined unorthodox perspective or a conservative view in which chiropractic subluxation/spinal dysfunction is considered an obstruction to human health.

Chiropractic school attended was found to be a significant predictor of orthodox versus unorthodox faction membership and professional practice characteristics in Canada [29].

Opinions of American (*n* = 1,024), Canadian (*n* = 76) and Mexican (*n* = 2) chiropractors varied on issues related to the historic conflict between broad scope (focusing on primary care or specialties dealing with a range of conditions beyond the spine) and focused (narrow) scope of practice (focusing on correcting subluxations in the spine to free the body’s self-healing capacity) [26]. Over 75 % of survey respondents favoured a broad scope of clinical services, practices, procedures and privileges. A similar proportion (75 %) believed that chiropractic ‘adjustment’ of the ‘vertebral subluxation’ was an effective treatment procedure for both musculoskeletal and selected visceral conditions. Representative samples of chiropractors in UK and in Canada indicated that between 50–60 % of respondents considered ‘science’ or EBP more important than traditional chiropractic beliefs or philosophy [27, 30]. Despite this, a similar proportion of respondents considered subluxation to be central to chiropractic intervention. In Canada, large discrepancies in scope of practice were reported by private clinic websites naming 159 distinct conditions treatable by chiropractic where professional association and college websites only identified 41 unique conditions [31].

Six studies reported on the influence of philosophical beliefs on practice behaviour (e.g., anti-vaccination, wellness and clinical management strategies, non-guidelines-based x-ray use) [18, 29, 32–34] and membership of professional associations [27]. Compared with those who viewed their scope of practice as narrow (historically referred to as ‘straight chiropractors’), ‘broad scope’ chiropractors tended to provide advice tailored to the patient’s condition (exercise, pain experience, and muscles involved for acute cases versus pain, diet, and calcium supplementation for chronic cases) [33].

##### Sub theme 2: Standards of care

A single study reported on Canadian chiropractors’ attitudes toward the development of standards of care [35]. However, the majority of survey respondents (74.6 %) indicated that they would be supportive of a national standard of care developed and implemented in collaboration with national and provincial organizations and chiropractic teaching institutions, specifically with a leadership role by the Canadian Chiropractic Association (CCA).

##### Sub theme 3: EBP attitudes and skills

Five studies found that chiropractors generally held positive attitudes towards EBP, and were interested in improving their EBP skills [36–40]. Nonetheless, application of EBP in clinical practice appears to be suboptimal, with only a small majority of American chiropractors (53 %) reporting that about half of their practice was based on research evidence [40]. Two studies surveyed Australian and German chiropractors respectively about the importance of research as a means to increase the credibility of the profession and to further explore inter-professional collaboration [37, 39]. Clinicians indicated that they were willing to support research efforts, mostly as participants (e.g., completing surveys) or to provide patient data [37].

#### Theme 2: Implementation of evidence-based practices and capacity building

Few studies have evaluated the impact of implementing EBP care approaches [41, 42]. One pragmatic RCT comparing an evidence-based treatment protocol with usual care for acute non-specific LBP found no important difference in outcomes [42]. However, the EBP treatment protocol generated results more rapidly than usual care and with fewer treatments. One uncontrolled (descriptive observational) study reported that protocols that were based on evidence-based CPGs produced better clinical outcomes with faster results, higher patient satisfaction, and at a lower cost than usual care [41]. A capacity building project enrolled 26 students in a 20-credit university postgraduate programme on EBP and research. While the programme raised awareness about EBP and research, and provided participants with the needed tools to use and implement EBP, none of the students developed a research protocol of sufficient quality to obtain a passing mark on the final assignment, and only two undertook a PhD programme [43].

II. Research Utilization (RU)

Twenty-one of the included studies (34.3 %) related to RU. Two themes emerged within the RU category: I) Guideline adherence (*n* = 13) and II) Sources of information generally used (*n* = 8).

#### Theme 1: Guideline adherence

Three cross-sectional studies [44–46] and one prospective cohort study [47] specifically related to adherence to diagnostic imaging guidelines. While training provided by teaching institutions on the use of radiography appeared to be evidence-based [44], clinicians’ awareness of available CPGs, intention to follow guidelines, and self-reported guideline adherence were generally low among chiropractors in Australia [45], but adequate among Norway chiropractors [46]. Interestingly, a smaller proportion of US chiropractors (19.6 %) did not adhere to guidelines for early magnetic resonance imaging of occupational low back pain compared to other medical providers (33.1 %) [47]. Guideline compliance for managing neck pain [48, 49], acute whiplash [50] and low back pain [51, 52] was also generally acceptable. For instance, three-quarters of chiropractors (76 %) believed that encouragement of maintaining normal activities, even in the presence of pain due to acute whiplash, was important to recovery [50]. Similarly, care delivered in individual chiropractic practices in the UK [53] and Northeastern Spain [54] were generally aligned with best practice. Two articles reported that a majority of clinicians in private practice used treatment not supported by current recommendations on nutrition [55, 56]. A best evidence synthesis on the management of low back pain concluded chiropractors had greater guideline adherence (73 %) than physiotherapists (PTs) (62 %) or medical practitioners (52 %) [57].

#### Theme 2: Frequency and sources of information accessed

Four studies indicated minimal use of evidence-based information sources (e.g., peer-reviewed journals, Cochrane Database of reviews, PubMed/Medline), with a preference for using lower quality and potentially misleading sources of information (websites, trade magazines, health magazines, books and colleagues) [32, 39, 55, 56]. While there was general agreement with teaching standards, chiropractors highlighted some discrepancies between the curricula (e.g., physical examinations procedures) and practitioners’ skills and knowledge use [58]. Online courses to acquire new knowledge seem to be the preferred mode of delivery of EBP information [55, 56]. A short training session on the use of online research literature appeared to increase providers’ belief about the usefulness of search engines such as PubMed [59]. Factors explaining the steady growth in health website recommendations to consumers by HCPs include the confidence that the website is a reliable source and that it complements care; patient’s requests and encouraging self-management, the potential to enhance the doctor-patient relationship and reduce consultation time [60]. Furthermore, recommending essential literature to inform evidence-based clinical practice has been advocated [61].

III. Knowledge Translation (KT)

Thirty-six of the 67 studies (53.7 %) related to KT. Articles corresponded to three themes: 1) Identifying knowledge-practice gaps (*n* = 12); 2) Assessing barriers and facilitators to knowledge use (*n* = 15); and 3) Selecting, tailoring, and implementing KT interventions (*n* = 9) (Fig. 6).

#### Theme 1: Identifying knowledge-practice gaps

Knowledge-practice gaps for chiropractors were identified in several studies on a number of topics including: risk factors for scoliosis progression [62]; establishing a prognosis for whiplash [63]; routinely using validated outcomes measures and patient-reported outcomes (other than pain and disability scales) [30, 64, 65]; offering neck pain patients treatments with limited support or conflicting evidence (e.g., ergonomics, relaxation techniques, patient education) [48, 49]; or offering acute whiplash patients non-evidence-based passive therapy, including traction or transcutaneous electrical nerve stimulation [50]. While clinicians reported routinely assessing physical impairments and pain, assessments of activity limitation and psychosocial function to help establish the prognosis were not commonly assessed [66].

Evidence-practice gaps were also identified regarding chiropractors’ attitudes about the management of public health issues [67–70]. In one US survey, although most respondents considered themselves as wellness-oriented providers, only 2 % of the participants had read the Healthy People 2010 national objectives, 27 % disagreed with the objectives and 29 % were unsure if their practice reflected the objectives [67]. While the majority of advice on health promotion did not significantly differ between American general medical doctors and chiropractors, only a third of patients with arthritis received advice to lose weight to alleviate their condition, whereas a higher proportion (approximately 60 %) were advised to increase exercise [68]. In North Carolina, USA, less than half of the surveyed 684 chronic back and neck pain patients were prescribed exercise (the desired evidence-based practice) after consulting GPs, chiropractors or PTs [69]. While a large majority of surveyed chiropractors in Great Britain reported adhering to evidence-informed practice (89 %), between 56-60 % of respondents discussed or monitored lifestyle issues such as smoking cessation or over-consumption of alcohol [70].

#### Theme 2: Barriers and facilitators to knowledge use

Lack of time, perceived lack of clinical evidence, lack of incentives, having graduated over 10 years ago, insufficient skills or confidence in locating, interpreting, critically appraising, and applying research findings to clinical practice were believed to be important barriers [40, 71–73]. Agreement with and motivations to follow guideline recommendations varied in the UK [74, 75], North America [72, 76] and Australia [36]. MSK practitioners (PTs, Chiropractors and Osteopaths) in the UK agreed that re-activation was a primary goal of LBP treatment [75]. Despite this however, chiropractors tended to recommend restricting daily activities. A follow-up survey on behaviour, beliefs, and attitudes to LBP management among these same MSK health disciplines suggested that many private practitioners do not see their role as directly intervening to reduce work absenteeism due to LBP [74]. An apparent barrier to guideline uptake is the limited awareness of best practice initiatives [77] and existing CPGs [76, 78]. Other barriers influencing uptake of CPGs [76] or electronic incident reporting systems [79] included beliefs about the consequences of following CPGs and fear of reporting incidents, influence from past training, peers and patients, concerns over providers’ social or professional role and identity, and self-confidence in managing patients [72, 76]. In a study on tobacco cessation training, interviewed practitioners identified perceived intrusiveness or potential patient social discomfort or alienation as barriers to uptake, preferring not to discuss tobacco use with new patients [80].

In contrast, one study determined that perceiving EBP as helpful in clinical decision-making increased the likelihood of chiropractors reporting using CPGs [36]. Facilitators of EBP uptake also included free online databases at work, online educational materials, and access to critical reviews and full-text articles [40]. Overall, findings suggest that guideline implementation could be strengthened if multifaceted interventions were used. There is a need for high quality EBP continuing education programmes and increased support from professional organizations to develop collegial support for EBP, and greater collaboration between researchers and practitioners to design clinically applicable research [81]. To help reduce risk of patient harm, Canyon (2013) recommends that chiropractic organizations address barriers to documenting harm by developing formal risk assessment strategies and improving their level of understanding of crisis management [82]. In one study chiropractors felt that CPGs should be developed specifically for chiropractors and not be widely applicable for all healthcare professions [35].

#### Theme 3: Selecting, tailoring, and implementing KT interventions

Nine studies examined the effect of educational interventions on process of care [83–87] and professional behaviour change [88–91]. Five of these articles targeted chiropractors only [83–85, 90, 91]. Although effect sizes were small to moderate, multifaceted (*n* = 4) and single KT interventions (*n* = 5) examining the effect of educational interventions (*interactive workshops with or without reminders, paper-based or online distribution of educational printed material, audit and feedback*) on proxy measures of behaviour change [83–87] and professional behaviour change [88–91] favourably shifted HCP beliefs and attitudes toward CPGs. These interventions also increased guideline adherence for managing spine pain or in using validated patient self-reported questionnaires. The few studies targeting chiropractors only [83–85] were underpowered, had a short follow-up [83, 84], had no control group and failed to use validated outcome measures [85, 91].

## Discussion

This scoping review reports on the current state of knowledge on evidence-based practice (EBP), research utilization (RU), and knowledge translation (KT) in chiropractic.

### Influence of chiropractors’ views on practice, health outcomes, and programmes and/or policy

The notion of two basic groups in chiropractic: “orthodox” and “unorthodox” through the early half of the 20th century has changed. A minority of clinicians (approximately 18 %) continue to hold a more traditional perspective [18, 92, 93]. Traditional views of chiropractic appear to negatively influence practice behaviour (e.g., anti-vaccination, non-guidelines-based x-ray use, and low adherence to patient education, work and activity recommendations) [18, 32–34] and adherence to professional associations [27]. In other words, 1 in 5 chiropractors may be delivering care that is not evidenced-based, potentially putting patients at risk of harm from communicable diseases and unnecessary radiation exposure and/or giving advice that delays patient recovery. While it is estimated that fewer than 5 % of initial patient consultations to chiropractors are for non-musculoskeletal disorders [94–96], there is currently minimal evidence to support chiropractic treatment for these conditions [97, 98]. Despite this, a recent review of websites of major chiropractic associations, colleges (*n* = 11) and commercial clinics (*n* = 80) across Canada suggests that over 30 % of included practices presented chiropractic as both an evidence-based profession in line with science and an alternative option for treating and addressing health concerns such as allergies, attention deficit disorder, bedwetting and premenstrual syndrome [99]. Such discourse and dissonance, and the high degree of variability in scope of practice across US states [100] and in other countries [101] are sources of confusion among members of the public, other health disciplines and policy makers [102].

Leaders in the profession and clinicians alike need to be aware of a moderate to strong association between HCPs’ attitudes and beliefs and their influence on the attitudes and beliefs, clinical management, and outcomes of patients with low back pain [34]. This is important in light of efforts in Canada and elsewhere to support health system reform that encourages prevention, inter-professional collaboration, evidence-based practice, patient-choice, and protection of the public interest [http://www.chiropractic.ca/about-cca/position-statements/]. Multilevel strategies involving professional chiropractic leaders, teaching institutions, researchers and other stakeholders are needed to help transform the culture of chiropractic toward one that is guided by EBP principles [103].

### Influence on EBP attitudes and barriers on uptake of EBP

Studies included in the current review found that chiropractors generally held positive attitudes towards EBP, and were interested in improving their EBP skills [36–40]. However, the use of evidence-based information sources is suboptimal [32, 39, 55, 56]. Recent studies in Canada [104] and in the US [81] support these findings. Positive relationships have been noted between research utilization and nurses’ beliefs and attitudes toward research and the different kinds of research utilization [9]. Further research is needed to establish if similar determinants apply in chiropractic.

### Uptake of best practice and guidelines

While guideline compliance for managing acute whiplash [50] and low back pain [51] was deemed acceptable, the application of EBP and adherence to imaging guidelines [40, 45, 83] and best practice [55, 56] appears suboptimal. Identified barriers and facilitators to using EBP and guidelines in chiropractic are similar to those reported in reviews of physicians and allied care providers [5, 105, 106]. Lack of time, perceived lack of clinical evidence, lack of incentives, motivation and agreement with CPGs, having graduated over 10 years ago, insufficient skills or confidence in locating, interpreting, critically appraising, and applying research findings to clinical practice are believed to be important barriers [36, 40, 71, 74–76]. In contrast, having a favourable attitude toward EBP [36], free online databases at work, online educational materials, and access to critical reviews and full-text articles [40, 55, 56] were perceived as facilitators of RU. Recently, the World Federation of Chiropractic (WFC) put forth such a reading list (http://www.wfcsuggestedreadinglist.com/#download). Other important resources on EBP can be found on the Canadian Chiropractic Guideline Initiative (CCGI) website with links to Cochrane reviews relevant to the scope of practice (www.chiroguidelines.org).

### Dissemination and implementation research

Although effect sizes were small to moderate, included studies evaluating the effect of educational interventions on process of care [83–87] and professional behaviour change [88–90] generally found that these strategies favourably shifted chiropractors’ beliefs and attitudes toward CPGs and increased guideline adherence. However, the research to support this is limited. Well-designed evaluation studies targeting chiropractors with larger sample sizes, longer follow-up, use of a control group and validated outcome measures are needed. Furthermore, the lack of consistency of effect in these studies may be related to the intervention strategies used. There is general agreement that implementation strategies are more effective if they address identified barriers to change [107], and that the effectiveness of strategies depends on the organizational context in which they are implemented [108]. Examples of theory-based KT interventions in chiropractic are those aiming to reduce spine imaging [109] or to increase use of multimodal care for neck pain [72] (Trial Registration: https://clinicaltrials.gov/, NCT02483091, registered 17 June 2015). Future dissemination and implementation efforts should aim to address knowledge-practice gaps identified in our review, including risk factors for scoliosis progression [62], establishing prognosis for whiplash [63], routinely using validated outcomes measures and patient-reported health outcomes (other than pain and disability scales) [30, 64, 65], the ongoing use of passive therapy to manage acute whiplash [50] and patient care concerned with public health issues [67–69].

### Relevance to end users (clinicians and patients)

Knowledge gained from this review provides a deeper insight on ways in which we can help end users engage in group discussions. For clinicians, strategies aimed at closing the evidence-practice gap can reduce inappropriate practice variations and improve process of care and patient outcomes. Chiropractors recognize the important of research to raise the credibility of the profession [37, 39] and are willing to support research efforts, mostly as participants (e.g., completing surveys) or by providing patient data [37]. Chiropractic Practice-Based Research Networks (PBRNs) in Canada [110], Europe [111], USA [112] and Australia [113] offer the opportunity for clinicians and patients to engage in meaningful clinical research to enhance the management of musculoskeletal care. PBRNs can facilitate recruitment and help retain participants, two essential but challenging aspects of clinical research [114, 115]. Furthermore, PBRNs may be ideal environments to increase understanding of barriers to professional behaviour change and to pilot test implementation of CPGs and best practices prior to scaling up interventions [110]. Details on projects from chiropractic PBRNs in Canada and Australia can be found elsewhere (http://www.chiroguideines.org and http://www.acorn-arccim.com/).

### Relevance to other practice, programmes and/or policy contexts

Interprofessional collaboration is an essential component for moving toward integrated health care [116, 117]. Findings from this scoping review indicate that chiropractic is actively engaged in interprofessional primary care research in the area of musculoskeletal disorders. As research capacity continues to grow in the chiropractic profession, it is envisioned that health service and KT research will increasingly influence practice, programmes and policy.

Chiropractic teaching institutions play a crucial role in promoting EBP/RU/KT with their students and alumni [118, 119]. To do so, academic programs should incorporate EBP content early in a program of study and “weave” it throughout the 4–5 years of professional education. Curricula [120] should be designed to foster the attributes known to support an EBP approach (i.e., attitudes, reflection, skills, knowledge, confidence, etc.) [15, 121]. EBP content should be situated in authentic contexts and using real life scenarios that resemble the types of cases graduates are most likely to encounter in their future clinical practice [120, 122]. Importantly, students should be scaffolded towards higher levels of performance of EBP and autonomy in applying EBP. This may be achieved by first expanding students’ knowledge base, then by focusing on the application of EBP for different and increasingly complex cases and finally, by fostering students’ ability to reflect upon and assess the outcomes of the EBP process. Faculty could promote EBP competencies by working closely with clinicians; together they can provide a more holistic view of EBP (i.e. from the classroom to its real life application) [123, 124]. Moreover, clinician “role models” and EBP “champions” are ideally positioned to influence the design and delivery of the EBP content in the classroom.

To increase the likelihood of successful change however, faculty members could benefit from continuing professional development; not only on the EBP process itself, but on how to teach and evaluate EBP (i.e., content knowledge and pedagogical content knowledge). The *Consortium for Evidence-Informed Practice Educators* (http://www.cameducatorsforeip.org/) and the *Academic Collaborative for Integrative Health* (http://www.accahc.org/) are examples of initiatives respectively aimed at providing leadership, training, and support to CAM educators in the principles, practice and teaching of Evidence-Informed Practice (EIP) and in cultivating partnerships and advancing interprofessional education and collaborative practice. Other important initiatives to promote the application of EBP include the development and dissemination of clinical practice guidelines and related tools by the *Council on Chiropractic Guidelines and Practice Parameters* (http://clinicalcompass.org/) and the *CCGI* (http://www.chiroguidelines.org). Together, these initiatives should facilitate the shift of the chiropractic profession toward EBP, which ultimately may translate in the delivery of safer and more effective patient centered care [125].

### Strengths, limitations and recommendations

This review complies with key steps outlined in the Joanna Briggs Institute manual for conducting scoping reviews, [126] including using an a-priori protocol, a search of several databases, consulting an experienced health sciences librarian to ensure our strategy was appropriate, keeping the search strategy unrestricted to study design, screening titles and abstracts followed by full-text screening for relevance using at least two independent reviewers, and using a standardized data abstraction form adapted to the focus of the review. Nonetheless, this review also has some limitations. The quality of included articles was not assessed as part of this scoping review. Further research is required into effective EBP training programmes for chiropractors to improve attitudes, skills and uptake of EBP. Research is also needed on whether specific elements of post-graduate (and undergraduate) training that can be identified as effective across the spectrum of chiropractors’ attitudes and beliefs about EBP to sustainably improve EBP knowledge, skills, attitudes and behaviours. There is a need for robust dissemination and implementation research to increase guideline adherence and improve patient health outcomes. Large integrated clinical and administrative databases can better our understanding of practice patterns and variations, incident reporting and safety measures in chiropractic, knowledge-practice gaps, and provide additional compelling evidence from chiropractic outcomes. One important barrier to conducting clinical research is recruitment. Despite an increased evidence base for methods to improve the response rates to postal questionnaires [127], there has been a steady downward trend in clinician’s response rates to surveys [128]. We estimated the response rate for 31 cross-sectional (survey) studies at less than 50 % (range 2-100 %), with greater participation from northern European countries compared to North America or Australia. However, this figure should be interpreted with caution considering several of the studies could not determine the number of invited subjects who actually opened the invitation letter or e-mail for surveys. Planned targeted dissemination strategies of the review findings include: presentations at key national conferences, partnerships with relevant stakeholders in the chiropractic community to identify strategies for information exchange, use of chiropractic opinion leaders to help disseminate findings and recommendations, creation of briefing notes highlighting key messages, recommendations, and action items, and posting on the Canadian Chiropractic Guideline Initiative (CCGI) website designed to share findings with end-users.

## Conclusion

Findings from this review suggest that the majority of chiropractors hold favourable attitudes towards, and beliefs about, EBP. However, much remains to be done for chiropractors to routinely apply evidence into clinical practice. Continuing education programmes should seek to increase chiropractors’ EBP skills. Clinicians are encouraged to consider newer resources aimed at facilitating the uptake of best practice and guidelines. Additional research is needed to identify determinants of, and barriers to, EBP and knowledge use among chiropractors and to test tailored dissemination and implementation strategies to increase adherence to best practice and guidelines and to improve patient health outcomes.

## Abbreviations

CAM, complementary and alternative medicine; CCA, Canadian Chiropractic Association; CCGI, Canadian Chiropractic Guideline Initiative; DC, Doctor of Chiropractic; EBP, evidence-based practice; HCP, Heath Care Professional; KT, knowledge translation; RU, Research Utilization.

## Additional files

Additional file 1: MEDLINE search strategy used to identify research articles. (PDF 102 kb) Additional file 2: Data Abstraction Table. (XLSX 213 kb) Additional file 3: Thematic analysis of articles*. (XLSX 24 kb)
