# Supplementary material for: Evidence-based practice, research utilization, and knowledge translation in chiropractic: a scoping review
Source: BMC Complement Altern Med. 2016 Jul 13;16:216. doi: 10.1186/s12906-016-1175-0 (PMC4944433; doi:10.1186/s12906-016-1175-0)
Supplement: Additional file 1: — MEDLINE search strategy used to identify research articles. (PDF 102 kb) [file 12906_2016_1175_MOESM1_ESM.pdf]

Research utilization, evidence-based practice and knowledge translation in chiropractic: A scoping review.

Supplementary file 1: MEDLINE search strategy used to identify research articles.

1	exp Clinical Competence/ or exp Information Dissemination/ or exp "Diffusion of Innovation"/ or exp Health Knowledge, Attitudes, Practice/ or organizational innovation/ or knowledge management/
2	exp Evidence-Based Practice/ or "evidence-based practice".ti,ab.
3	Practice Guidelines as Topic/ or exp Guideline Adherence/
4	Professional Practice/ or exp Physician's Practice Patterns/ or practice pattern*.mp.
5	Quality improvement.mp. or Quality Improvement/
6	(organi*ational adj3 innovation).ti,ab.
7	(complex adj3 intervention).ti,ab.
8	(scholarship adj3 practice).ti,ab.
9	(practice adj3 development).ti,ab.
10	(evidence-informed adj (healthcare or health care or decision making)).ti,ab.
11	(guideline* adj2 (introduc* or issu* or impact* or effect* or distribut* or adher* or compliance* or utili*ation or "use" or uptake or diffusion or transfer* or implement* or translat* or disseminat* or adopt*)).ti,ab.
12	((research or knowledge or evidence) adj2 (uptake or "use" or diffusion or disseminat* or utili*ation or transfer* or translat* or implement* or adoption)).ti,ab.
13	"research into practice".ti,ab.
14	"evidence into practice".ti,ab.
15	"knowledge to action".ti,ab.
16	"know do gap".ti,ab.
17	(knowledge adj (mobilization or exchange)).ti,ab.
18	(belief* or attitude*).ti.
19	(benchmark* or bench mark*).ti,ab.
20	benchmarking/
21	1 or 2 or 3 or 4 or 5 or 6 or 7 or 8 or 9 or 10 or 11 or 12 or 13 or 14 or 15 or 16 or 17 or 18 or 19 or 20
22	Manipulation, Chiropractic/ or Chiropractic/
23	chiropract*.tw.
24	chiroprax*.tw.
25	spinal manipulative therap*.mp. or spinal manipulation.tw.
26	Manipulation, Spinal/
27	22 or 23 or 24 or 25 or 26
28	21 and 27
